# Inpatients in substance use treatment with co-occurring psychiatric disorders: a prospective cohort study of characteristics and relapse predictors

**DOI:** 10.1186/s12888-023-04632-z

**Published:** 2023-03-09

**Authors:** Helle Wessel Andersson, Mats P. Mosti, Trond Nordfjaern

**Affiliations:** 1 Department of Research and Development, Clinic of Substance Use and Addiction Medicine, St. Olavs University Hospital, Pb 3250 Sluppen, Trondheim, 7006 Norway; 2 Department of Psychology, Norwegian University of Science and Technology, Trondheim, Norway

**Keywords:** Substance related disorders, Psychiatric comorbidity, Treatment outcome, Cannabis use, Motivation

## Abstract

**Background:**

The characteristics of substance use disorder (SUD) inpatients with co-occurring psychiatric disorders (COD) have been scantly described in the extant literature. This study investigated psychological, demographic and substance use characteristics in these patients, along with predictors of relapse 3 months post-treatment.

**Methods:**

Prospective data from a cohort of 611 inpatients were analyzed for demographics, motivation, mental distress, SUD diagnosis, psychiatric diagnoses (ICD-10) and relapse rate at 3 months post-treatment (retention rate = 70%).

**Results:**

Compared to patients without COD (n = 322), those with COD (n = 289) were younger, had higher mental distress, lower education and higher likelihood of no permanent residence. The relapse rate was also higher in patients with COD (39.8%) relative to patients without COD (26.4%) (OR = 1.85, 95% CI: 1.23–2.78). The relapse rate was particularly high for patients with COD who were diagnosed with cannabis use disorder (53.3%). Multivariate analysis revealed that among patients with COD, relapse was more likely for individuals with a cannabis use disorder (OR = 2.31, 95% CI: 1.34-4.00), and less likely for older ages (OR = 0.97, 95% CI: 0.94-1.00), females (OR = 0.56, 95% CI: 0.33–0.98) and for those with higher intrinsic motivation (OR = 0.58, 95% CI: 0.42–0.81).

**Conclusion:**

This study showed that among SUD inpatients, those with COD had relatively persistent high levels of mental distress and an increased risk of relapse. Enhanced measures aimed at COD patients’ mental health problems during the inpatient stay, along with close and personalized follow-up after discharge from residential SUD treatment may reduce the probability of relapse in this group.

## Background

Persons in inpatient treatment for substance use disorders (SUD) have extensive service needs [1] linked to a complexity of symptoms [2, 3], including psychological and psychiatric burden [4–6]. In particular, there is a high prevalence of co- occurring psychiatric disorders (COD) (i.e. the co-occurrence of SUD with another psychiatric disorder) in SUD inpatient treatment settings, with rates around 50% or above [7–9]. The combination of substance use and psychiatric disorders may complicate shift in substance use behavior [10] and worsen treatment prognosis [11]. This combination is also associated with more severe features, such as more severe psychiatric symptoms, substance use, and relapse [12–15]. Despite this, little is known regarding the demographic, psychological and clinical characteristics (e.g. types of SUD diagnoses and psychiatric diagnoses) of inpatients with COD, and the extent to which these factors have an impact on their treatment outcome.

A review of studies reporting the prevalence of COD within diverse SUD treatment settings in Australia [16] concluded that most studies did not analyze demographic characteristics among patients with COD. Regarding inpatient SUD treatment settings, the few studies that compared the demographic characteristics of patients with - and without COD, revealed a predominance of females among the former group of inpatients [7, 8]. It was also indicated that the age distribution was roughly the same across the two patient groups [7, 12]. Results regarding education level were mixed. For example, one study found that the education level of inpatients with and without COD was comparable (7), while another study (12) reported that patients with COD had more education than those without COD.

Studies investigating psychological characteristics among patients with and without COD consistently showed that those with COD reported higher levels of general mental distress, [12–14, 17]. Anxiety and depression are the two most frequently co-occurring psychiatric disorders among SUD patients [16]. One study showed that these disorders occurred particularly frequently among female inpatients with COD [8]. An unaddressed research question in the literature is whether the prevalence of COD varies with the specific type of SUD [16]. As far as we know, the study by Bergman et al. (7), suggesting that patients with COD were more likely diagnosed with alcohol use disorders and polysubstance use, has been the only study to investigate types of drugs involved in COD within an inpatient SUD treatment setting. However, their study was restricted to young adult patients (18–25 years), and did not use objective diagnostic criteria, limiting the generalizability of findings.

Our prior research among a general SUD inpatient sample showed that having a co-occurring psychiatric disorder was associated with an increased relapse risk [15]. However, the specific relapse predictors among inpatients with COD has not been investigated previously. Previous research on general SUD treatment samples has identified both demographic and psychological factors that may have an impact on SUD treatment outcome (including treatment dropout and relapse). These variables included younger age [15, 18, 19], lower education, and unstable living arrangements [20], whereas previous research on putative gender differences in SUD treatment outcomes revealed mixed results [21–23]. Among psychological variables, both baseline motivation [24–26] and baseline mental distress level (i.e. symptoms of anxiety/depression) [24, 27–29] have been found to be associated with SUD treatment outcomes.

Whether different types of SUD diagnoses influence the treatment outcome of patients with COD has not been sufficiently investigated. While analyses of data from different SUD treatment programs in USA [30] suggested higher risk of treatment non-completion among patients with COD admitted for alcohol and cannabis use, previous research did not examine whether the risk of post-treatment relapse among COD inpatients varied with particular types of SUD diagnoses.

There is a need for updated and extended knowledge about the characteristics of SUD inpatients with COD, and the factors associated with their treatment outcome. This study will advance existing research by investigating such factors among a non-selected inpatient SUD treatment sample. The aims were: (1) To examine demographic, psychological, and diagnostic characteristics of COD inpatients; (2) To investigate the risk for post-treatment relapse rates according to types of SUD diagnoses; (3) To identify patient characteristics associated with post-treatment relapse to substance use.

Based on previous research from general SUD treatment samples, we hypothesized that patients with COD would be more marginalized in terms of mental distress and post-treatment relapse rate. We also hypothesized that higher levels of intrinsic motivation for changing substance use behavior would have a moderating effect on relapse risk.

## Methods

### Setting and design

We used data from a prospective cohort study of patients admitted to an inpatient SUD treatment stay at five treatment centers in central Norway. Patients were recruited in the period from September 2014 to May 2016. Study participation involved providing demographic, substance use and health information, from a questionnaire and the electronic medical record, as well as a follow-up interview 3 months after the end of the inpatient stay. The Regional Ethical Committee for Medical Research in Norway reviewed the study protocol and approved the study (#2013/1733). In accordance with the Declaration of Helsinki, those who agreed to participate gave their signed consent. Dedicated research staff affiliated with the study were responsible for continuously recruiting patients admitted for inpatient treatment at the five clinics. The only exclusion criteria were persons judged mentally incapable of giving their signed consent for participation. Of 728 eligible patients, 611 (84%) consented to participate (see 15, 24 for more details).

### Variables

#### Demographics

Demographic characteristics included age at treatment entry, gender, education level (low: 10 years primary and secondary education or less, or medium/high: high school/vocational school or more) and housing situation (living in owned home/rented housing, or in an unstable living arrangement, including living with family or friends).

#### Motivation

Five items concerning intrinsic motivation for changing personal substance use were used to measure baseline motivation. The items were obtained from the Circumstances, Motivation Readiness and Suitability instrument (CMRS) [31]. The patient responses were rated on a scale ranging from 1 (completely disagree) to 5 (totally agree), (α = 0.83).

#### Anxiety/depression symptoms

The Norwegian version [32] of the self-reported Hopkins Checklist-10 (HSCL-10) [33] was used to measure mental distress at baseline and 3-month follow-up. The patients reported how frequently they had experienced symptoms of anxiety and depression in the past 7 days on a 1–4 scale (1 = not at all; 4 = extremely) (baseline α = 0.89; follow-up α = 0.91).

#### Diagnoses

The SUD diagnoses and psychiatric diagnoses according to ICD-10 criteria, were made by a medical specialist or clinical psychologist using standardized clinical interviews and tools.

For the purpose of the current study, information on the dependence-level SUD diagnosis and any co-occurring psychiatric diagnosis was obtained from the medical record. The following binary SUD diagnosis (1 = presence, 0 = absence) were included in analyses: Alcohol use disorder (F10); Opioid use disorder (F11); Cannabis use disorder (F12); Sedatives use disorder (F13); Stimulant use disorder (F15). The psychiatric diagnoses were grouped into the following binary variables (1 = presence, 0 = absence): Mood disorders (F30-F39); Anxiety disorders (F40-F49); Personality disorders (F60-F69); ADHD (F90-F90.0), and other psychiatric diagnoses.

#### Treatment outcome: relapse to substance use

The 3 month post-treatment follow-up telephone interview asked about the use of alcohol or drugs during the last four weeks. Patients indicated how often they had used alcohol/drugs during this period, with the following response options: “less than once a week,” “approximately weekly,” “2–4 times a week,” “daily or almost daily”. We defined relapse as return to regular use [15], thus those who reported using alcohol or drugs 2–4 times or more per week were categorized as having a relapse. The interview also enquired about any contact (yes/no) with outpatient SUD treatment services; and/or a community mental health and addiction health care provider; and/or readmission to SUD inpatient treatment. A small number of patients who reported readmission to SUD treatment was included in the relapse group (see also [34].

### Statistical procedures

Statistical analysis included descriptive statistics for the prevalence of co-occurring psychiatric diagnoses in the total sample, and according to types of SUD diagnoses. We compared the characteristics of patients with - and without COD using proportion tests and independent samples t-tests. The prevalence of types of CODs was examined for the following psychiatric disorders: anxiety (F40-F49); mood (F30-39), ADHD (F90-90.9); personality disorder (F60-69); multiple CODs. Gender differences in the prevalence of each types of CODs were examined using bivariate logistic regression analysis. Bivariate logistic regression analyses were also undertaken to investigate factors associated with relapse. Repeated-measures generalized logistic mixed modeling (GLMM) with a diagonal covariance matrix was used to assess the multivariate association of demographic (age, gender, education), psychological (motivation, mental distress) and types of SUD diagnoses with relapse at 3 month follow-up. The analysis accounted for the prospective nested nature of the data structure (i.e., the same patients nested over time). Since mental distress was measured at two time points (baseline and follow-up), this variable was entered as a time-varying covariate accounting for variation in mental distress across the study period. Variables indicating the center where the patients were treated (unit 1–5) and the length of stay (number of days) were included in the multivariate models to control for any treatment- related differences in relapse rates. We did not incorporate the treatment center variable as a random effect in the analysis due to the small number of patients at each treatment center, which made it complicated to account for the variance of treatment center as a random effect due to the substantial risk of Type II error. The variance inflation factors were < 2 for all independent variables, indicating that multicollinearity was not a concern [35]. We ran the GLMM analyses separately for patients with and without COD. SPSS 28 was used for statistical analyses.

### Results

#### Participants

The sample comprised 611 patients who were included in the prospective cohort study, of whom 289 patients (47.3%) had at least one co-occurring psychiatric diagnosis (F20-F99).

In total, 426 of the patients participated in the follow-up interview 3 months after discharge from treatment (70%), of whom 206 (48.4%) were patients with COD. The follow-up response rate was similar for patients with COD (71.3%) and those without COD (68.3%). Among patients with COD, those who did not respond were more likely younger (OR = 2.54, p = 0.002), with lower education level (OR = 1.77, p = 0.035), and less likely to have an alcohol use disorder (OR = 0.588, p = 0.053). Among patients without COD, those who were lost for follow-up appeared more likely younger (OR = 2.157, p = 0.002), and without a permanent housing situation (OR = 1.694, p = 0.042). About half of those who were reached at follow-up (n = 227) reported they had been in contact with SUD outpatient treatment services during the last month. Slightly fewer patients (n = 194) reported contact with a community health provider. The probability of contact with outpatient SUD services was somewhat higher for patients with COD (58.3%) than for patients without COD (48.6%) (p = 0.047). There was no difference between the groups regarding any contact with community mental health and addiction services.

#### Demographic, psychological and SUD diagnostic characteristics of patients with COD

Table 1 shows the demographic and clinical characteristics of the total sample, and for patients with COD compared to those without. Patients with COD were younger than those without COD, and more patients in the COD group had a lower education level. In addition, those with COD were more likely to have an unstable housing arrangement, and to have higher baseline and follow-up levels of mental distress. We found a significant reduction in mental distress at follow-up in both groups of patients. Moreover, the baseline motivation to change substance use behavior was as high in patients with COD as among patients without COD. With respect to types of SUD diagnoses, the results revealed that patients with COD were less likely than patients without COD to have an alcohol use disorder (F10). Patients with COD were comparatively more likely to have each of the illicit drug use disorders (i.e. cannabis use disorder, sedatives use disorder, opiate use disorder and stimulant use disorder), and polysubstance use. The data also showed that patients with COD had significantly longer treatment stays.

**Table 1:** Sample characteristics of patients with COD compared to patients without COD.

Variables	Total (N = 611)		COD (n = 289)		Without COD (n = 322)		COD versus without COD	
	N^a^	% or mean (SD)	n	% or mean (SD)	n	% or mean (SD)	p-value	Effect size
Age (years) at intake	610	30.0 (13.9)	288	32.9 (11.6)	322	42.7 (14.1)	< 0.000	0.759
Female	175	28.7	91	31.6	84	26.2	0.139	0.060
Education level low	189	32.6	109	39.5	80	26.3	< 0.001	0.140
Unstable housing arrangement^1^	210	35.1	117	41.2	93	29.6	0.003	0.121
Motivation	610	4.25 (0.77)	289	4.26 (0.78)	321	4.24 (0.75)	0.663	0.027
Mental distress baseline	611	2.15 (0.71)	289	2.30 (0.69)	322	2.02 (0.70)	< 0.001	0.403
Mental distress follow-up^2^	425	1.93 (0.74)	206	2.07 (0.73)	219	1.79 (0.72)	< 0.001	0.386
Improved mental distress^3^	425	0.22 (0.79)	206	0.22 (0.79)	219	0.23 (0.80)	0.852	0.013
Length stay (days)	611	93.8 (79.6)	289	112.2 (92.2)	322	77.2 (61.9)	< 0.001	0.446
*SUD diagnoses* ^*4*^	585	95.7						
- Alcohol use F10	345	59.0	132	48.4	213	68.3	< 0.001	0.202
- Opioid use F11	111	19.0	64	23.4	47	15.1	0.010	0.107
- Cannabis use F12	216	36.9	128	46.9	88	28.2	< 0.001	0.193
- Sedative use F13	170	29.1	99	36.3	71	22.8	< 0.001	0.148
- Stimulant use F15	188	32.1	105	38.5	83	26.6	0.002	0.127
- Alcohol use only	229	39.1	62	22.7	167	53.5	< 0.001	0.315
- Polysubstance use^5^	134	21.9	77	28.2	57	18.3	0.004	0.118

^1^Reference: Owned or rented residence. ^2^ For both patients with and without COD, the mean score of mental distress was lower at follow up (t = 41.15, df = 205, p < 0.001, and t = 36.66, df = 218, p < 0.001, respectively). ^3^ Calculated by subtracting the mean follow-up score from the mean baseline score. ^4^ More than one diagnosis could be registered for each patient. The most common two-substance combination was cannabis use and stimulant use (n = 113).^5^ Three or more SUD diagnoses

Notes: Percentages in valid percent. Categorical variables presented as valid percentages (%); continuous variables presented as mean (SD). Effect size was measured using Cohen’s d and Cramer’s V, as appropriate

#### Prevalence of co-occurring psychiatric disorders

The proportion of patients with COD was not significantly different for male (45.4%) and female (52.0%) patients (p = 0.139). The prevalence rates for the types of co-occurring psychiatric diagnoses are shown in Table 2. Among patients with COD, anxiety (22.9%) and mood disorders (17.3%) were the two most common psychiatric disorders. About one in five had more than one COD (21.4%), with a higher prevalence rate of multiple CODs among females (30.3%) than males (18.0%). Having anxiety disorders were significantly more prevalent among females (30.3%) than males (19.8%).

**Table 2:** Prevalence of co-occurring psychiatric disorders

	Total (N = 611)		Females (N = 175)		Males (N = 434)		Females versus males^ref^	
	N	%	N	%	N	%	OR	p-value
Without COD	322	52.7	84	48.0	237	54.6		
With COD	289	47.3	91	52.0	197	45.4	1.30	0.139
*Psychiatric diagnoses* ^1^								
- Anxiety disorders F40-49	140	22.9	53	30.3	86	19.8	1.76	**0.005**
- Mood disorders F30-39	106	17.3	36	20.6	70	16.1	1.35	0.191
- ADHD F90.0-F90.9	79	12.9	21	12.0	58	13.4	0.88	0.650
- Personality disorders F60-69	70	11.5	27	15.4	43	9.9	1.66	0.053
- Multiple CODs	131	21.4	53*	30.3	78	18.0	1.98	**< 0.001**

^1^ Other psychiatric diagnoses (n = 17) included Schizophrenia, F20-F29 (n = 8); Behavioral syndromes associated with physiological disorders and physical factors (n = 16); Mental retardation, F70-F79 (n = 6); Pervasive and specific developmental disorders, F80-89 (n = 16); Behavioral disorders, F91-F98 (n = 8)

#### Relapse rates and factors associated with relapse

Table 3 shows the results of comparisons of relapse rates for patients with and without COD and the risk of relapse (ORs) associated with different types of SUD diagnoses.

**Table 3:** Relapse rates according to types of SUD diagnoses. Patients with COD compared with patients without COD.

Types of SUD diagnoses	Mental disorder status	Total	Follow-up	Relapse^1^	OR^2^	95% CI
		N	N	%		
Total	COD	289	206	39.8	1.85**	1.226;2.782
	without COD	322	220	26.4	1.00	
Alcohol use	COD	132	102	37.3	1.68	0.978;2.877
	without COD	213	153	26.1	1.00	
Opioid use	COD	64	41	43.9	2.00	0.745;5.367
	without COD	47	32	28.1	1.00	
Cannabis use	COD	128	90	53.3	2.12*	1.083;4.160
	without COD	88	60	35.0	1.00	
Sedative use	COD	99	73	46.6	2.08	0.939;4.600
	without COD	71	44	29.5	1.00	
Stimulant use	COD	105	70	44.3	2.32*	1.058;5.103
	without COD	83	51	25.5	1.00	

^1^ Valid percentages. Proportion of patients who relapsed within each category

^2^ Unadjusted odds ratio

Boldface indicate statistically significant ORs *p < 0.05; **p < 0.01.

Among patients with COD, the post-treatment relapse rates varied from 37% for those with alcohol use disorders to 53% for those with cannabis use disorders. Among patients with COD, those with cannabis or stimulant use disorder were above two times more likely to relapse, compared to patients without COD having these SUD diagnoses, respectively.

The results of bivariate and multivariate associations with relapse are shown in Table 4. The multivariate analysis revealed that among patients with COD, those with a cannabis use disorder had an elevated risk of relapse (OR = 2.31), whereas older ages (OR = 0.97), female gender (OR = 0.56) and higher levels of baseline motivation to change substance use behavior (OR = 0.58) were factors associated with a reduced relapse risk.

Among patients without COD, increased risk of relapse was predicted by having lower education level (OR = 2.87), cannabis use disorder (OR = 2.24) and higher time-varying mental distress (OR = 2.22).

**Table 4:** Bivariate and multivariate analysis of relapse predictors. Patients with COD and patients without COD.

Variables	Unadjusted OR	95% CI		Adjusted^1^ OR	95% CI
*COD*					
- Age	0.96***		0.931;0.981	0.97*	0.942;0.998
- Gender^a^	0.68		0.367;1.251	0.56*	0.326;0.976
- Education^b^	1.89*		1.041;3.417	1.48	0.899;2.432
- Unstable housing arrangement^C^	0.53*		0.299;0.944	0.86	0.505;1.47
- Motivation	0.55**		0.377;0.794	0.58**	0.415;0.811
- Alcohol use^d^	0.77		0.433;1.360	1.47	0.862;2.514
- Opioid use^e^	1.21		0.601;2.419	1.68	0.878;3.216
- Cannabis use^f^	2.77***		1.535;4.982	2.31**	1.336;4.008
- Sedative use^g^	1.51		0.839;2.721	0.99	0.583;1.677
- Stimulant use^h^	1.29		0.714;2.337	1.13	0.668;1.899
- Mental distress	1.25^2^		0.832;1.879	1.41^3^	0.985;2.006
*Without COD*					
- Age	1.00		0.975;1.018	1.00	0.990;1.006
- Gender^a^	1.25		0.643;2.448	1.16	0.645;2.101
- Education^b^	2.30*		1.155;4.589	2.87**	1.516;5.447
- Unstable housing arrangement^C^	1.23		0.601;2.500	1.39	0.696;2.76
- Motivation	0.97		0.653;1.429	0.85	0.609;1.184
- Alcohol use^d^	0.87		0.449;1.668	1.16	0.488;2.735
- Opioid use^e^	1.07		0.463;2.472	0.41	0.155;1.057
- Cannabis use^f^	1.72		0.900;3.278	2.24*	1.086;4.627
- Sedative use^g^	1.17		0.565;2.440	1.213	0.522;2.822
- Stimulant use^h^	0.91		0.442;1.853	0.45	0.194;1.053
- Mental distress	1.621* ^2^		1.066;2.464	2.22*** ^3^	1.577;3.133

Notes. Boldface indicate statistically significant ORs.*p < 0.05; **p < 0.01; ***p < 0.001

References: ^a^.Male (0). ^b^ Reference: Higher educational level (0). ^C^ Reference: Owned residence (0). ^d−h^ Reference: other SUD diagnoses (0)

^1^Results of generalized mixed model analysis

^2^ Baseline measurement

^3^ Time-varying (baseline/follow-up)

## Discussion

The current study has provided updated and extended data on the characteristics of SUD patients with co-occurring psychiatric disorders, by investigating a large non-selected inpatient SUD treatment sample. The study also adds a through comparison of post-treatment relapse risk and associated factors among inpatients with and without COD.

The high prevalence of co-occurring psychiatric disorders found in our sample corresponds to previous research comprising inpatient SUD treatment populations [7–9]. Also as expected [8, 16], anxiety and depression were the most frequently occurring disorders.

As hypothesized, our data showed that patients with COD were younger, and appeared to be more socially and socio-economically deprived. The fact that patients with COD had longer inpatient treatment stays compared to patients without COD probably reflects a more challenging symptom profile and management in these patients (10, 11).

Previous research among SUD inpatients has not explored the socio-demographic characteristics of patients with COD, however the current finding correspond to reports from mental health treatment settings [36]. We did not find a higher prevalence of COD among females as was suggested in previous work [8, 30, 37]. It is noteworthy, however, that females had twice the risk of multiple co-occurring psychiatric diagnoses compared to males, and that particularly anxiety disorders occurred more frequently in females. The current results accords with research suggesting that females with substance dependence may have more complex mental health issues than males [38, 39].

In line with prior research including SUD inpatient treatment samples [12, 13, 17] patients with COD had indications of persistently poorer psychological functioning in terms of higher levels of mental distress, as compared to patients without COD. This finding underscores the need for SUD treatment providers to attend to mental health problems among patients with COD, and also alludes to the importance of available and accessible community mental health services upon discharge from the inpatient stay. An important area for future research is whether the treatment provided for patients with COD is in accordance with the recommendations in national treatment guidelines [40], which includes mapping and assessment of mental health, and the use of evidence-based interventions, such a cognitive behavioral therapy [41] and motivational interviewing [42]. In this context, it would also be interesting to examine the effect of integrated dual disorder treatment (IDDT) [43] which is suggested to be a promising approach for the treatment of patients with co-occurring SUD and psychiatric disorders [42].

Little research has been conducted to investigate whether the prevalence of COD among inpatients in SUD treatment varies with the type of SUD diagnosis. The current study showed that patients with COD had a distinct drug use profile. Contrary to Bergmann et al. (7), who reported that patients with COD were characterized by having an alcohol use disorder, we found that the proportion of patients who were dependent upon illicit drugs, particularly cannabis use disorders, was higher among patients with COD relative to patients without COD. The divergent results may be attributed to methodological differences between the studies. For example, while Bergmans’s study (7) was restricted to young adult SUD inpatients, our study included a relatively large and heterogeneous COD inpatient sample in terms of age and SUD diagnoses.

In accordance with our previous research conducted among a general SUD treatment sample of illicit drug users (15), the current study indicated a higher relapse rate among patients with COD as compared to those without COD. The present data showed that the relapse rate was particularly high among those with a cannabis use disorder, among whom more than 50% of the patients with COD reported a relapse at follow-up.

The multivariate analysis indicated that among the demographic variables included in the model, age and gender emerged as uniquely associated with relapse in patients with COD. Whereas previous studies did not evaluate predictors for relapse in patients with COD specifically, the current finding aligns with previous research within the general SUD inpatient population demonstrating an association between younger age and adverse SUD treatment outcomes, including treatment dropout (18) and relapse (15). Knowledge about the characteristics of the most vulnerable SUD patient groups is essential for the development of targeted preventive measures for relapse. Thus, future research among patients with COD may want to focus specifically on the youngest patient group, in an effort to reveal the extent to which they receive adequate treatment, including accommodating their needs for psychiatric and psychological treatment. We found that the female patients with COD were less prone to relapse than their male counterparts. The psychosocial and social implications of substance use differ among males and females (23), and may account for the mixed findings among previous studies that considered gender difference in relapse rate (e.g. 21, 22). Further studies, with sufficient statistical power, should examine factors that may moderate the association between gender and treatment outcome among patients with COD. For example, further research could investigate whether the lower risk of relapse in females could be explained by better access to protective factors such as social networks and community health services.

Our finding concerning the reduced relapse risk associated with higher baseline motivation was expected, and it is in accordance with a previous study reporting a positive association between high initial readiness for substance use change and treatment outcome (i.e. retention) among inpatients with COD [26]. Our results suggest that baseline motivation may be a particularly relevant risk factor of relapse among these patients. The association between baseline motivation and relapse among patients with COD may be due to underlying moderating characteristics, such as these patients’ tendency of poor SUD treatment attendance and engagement [10, 11]. Nonetheless, the current data suggest a potential of motivational enhancement interventions in contributing to reduced relapse risk among patients with COD.

Higher mental distress level (i.e. level of anxiety/depression symptoms) did not influence the relapse risk among patients with COD. This finding suggests that problems associated with having a psychiatric diagnosis, such as adverse social [36] and socio-economic characteristics [44], rather than the associated psychological symptoms per se, make these patients more prone to relapse. Studies of general SUD treatment samples have found that higher mental distress (i.e. anxiety/depression symptoms) is associated with adverse treatment outcomes [24, 27–29] as was also found in the current study among the patient group without COD. The higher predictive value associated with mental distress among patients without COD may be associated with untreated anxiety/depression symptoms, and/or that health care providers are insufficiently attentive to their mental health care needs following an inpatient treatment stay.

A unique relapse risk associated with cannabis use disorder was equally high in patients with COD, as in those without COD. Previous research has shown that among SUD patients, those with cannabis use disorders are typically below 25 years [27, 45]. These young adult SUD patients may have a distinct profile [27, 46], including the presence of more adverse psychosocial risk factors [47]. Thus, associated unmeasured background factors may have contributed to the increased relapse risk found among patients with cannabis use disorders in the present study. Patients with cannabis use disorders make up a large proportion of the SUD inpatient sample. It is possible that cannabis holds a lower perceived severity profile among patients, at least compared to other substances, which may contribute to a higher relapse risk as the potential severity of consequences might be perceived as less harmful. Studies to come could investigate the role of substance risk perception on relapse rates. Moreover, there is a need for quality research studies to identify the factors that contribute to the increased relapse risk among these patients.

### Strengths and limitations

Strengths of this study include the prospective study design and the relatively large SUD inpatient sample size. The sample size enabled a comparison of patients with and without COD with respect to several clinical and demographic factors and follow-up data on relapse rates and associated risk factors. Although the use of objective diagnostic criteria (ICD-10) for both SUD and psychiatric disorders represents a methodological strength of this study, as this is the most relevant source of diagnostic information available for clinicians, we cannot rule out the possibility of both over - and under-diagnostics [48]. The study has some other limitations that should be considered. A limited number of sociodemographic variables were included. Other sociodemographic variables, potentially predictive of SUD treatment outcome, such as for example employment status [49, 50] and previous criminal problems [51] were not available in this study. We used self-report data of relapse as an outcome variable. The accuracy of self-report data on relapse may be questioned. However, several studies have found satisfactory test-retest reliability regarding substance use [52–54]. Nonetheless, the use of laboratory tests to show relapse to substance use could have provided more accurate data. About 30% of the COD sample were lost for follow-up assessments. This rate was just as high for patients without COD, and was otherwise in line with other SUD follow-up studies [55]. Those who were lost for follow-up were more likely than those who retained to manifest social marginalization (i.e. low education, unstable housing situation). Thus, the current patient sample who were assessed for follow-up may be somewhat biased towards the less marginalized, and we cannot rule out that other relapse predictors could have occurred if also the more marginalized patients responded to the follow-up interview.

## Conclusion

Our study showed that inpatients with COD had relatively persistent high levels of mental distress and an increased risk of relapse, which was particularly evident among those with cannabis use disorder. Close and personalized follow-up of patients with COD after discharge from residential SUD treatment may reduce the probability of relapse. Our work also suggest that intrinsic motivation for changing personal substance use may have a moderating effect on relapse risk in patients with COD. Thus, evaluating intervention efforts to strengthen intrinsic motivation as a strategy to prevent relapse constitute an important area for future research. Moreover, we suggest that our findings may provide a basis for future research to assess whether SUD treatment provided for patients with COD, including both specialized outpatient and community-based follow-up health services, are sufficiently geared towards these patients’ psychosocial treatment needs.

## Data Availability

The data that support the findings of this study are available from author HWA upon reasonable request.

## List of abbreviations

SUD: substance use disorders
COD: co-occurring psychiatric disorders
ICD-10: International Statistical Classification of Diseases and Related Health Problems 10th version
OR: odds ratio
CI: confidence interval
ADHD: Attention Deficit Hyperactivity Disorder
GLMM: generalized logistic mixed modeling
IDDT: Integrated dual disorder treatment
